# SARS-CoV-2 interacts with platelets and megakaryocytes via ACE2-independent mechanism

**DOI:** 10.1186/s13045-021-01082-6

**Published:** 2021-04-29

**Authors:** Shu Shen, Jingyuan Zhang, Yaohui Fang, Sihong Lu, Jun Wu, Xin Zheng, Fei Deng

**Affiliations:** 1 State Key Laboratory of Virology, Wuhan Institute of Virology, Chinese Academy of Sciences, Hubei Province, Xiaohongshan 44#, Wuchang District, Wuhan City, 430071 People’s Republic of China; 2 National Virus Resource Center, Wuhan Institute of Virology, Chinese Academy of Sciences, 430071 Wuhan, People’s Republic of China; 3 Department of Infectious Diseases, Union Hospital, Tongji Medical College, Huazhong University of Science and Technology, Hubei Province, Jiefang Avenue 1277#, Jiangan District, Wuhan City, 430022 People’s Republic of China

**Keywords:** COVID-19, SARS-CoV-2, Platelets, Platelet activation, ACE2, Alternative receptors

## Abstract

Evidence suggests that platelets may directly interact with SARS-CoV-2, raising the concern whether ACE2 receptor plays a role in this interaction. The current study showed that SARS-CoV-2 interacts with both platelets and megakaryocytes despite the limited efficiency. Abundance of the conventional receptor ACE2 and alternative receptors or co-factors for SARS-CoV-2 entry was characterized in platelets from COVID-19 patients and healthy persons as well as human megakaryocytes based on laboratory tests or previously reported RNA-seq data. The results suggest that SARS-CoV-2 interacts with platelets and megakaryocytes via ACE2-independent mechanism and may regulate alternative receptor expression associated with COVID-19 coagulation dysfunction.

**Supplementary Information:**

The online version contains supplementary material available at 10.1186/s13045-021-01082-6.

## To the editor

**Fig. 1:**
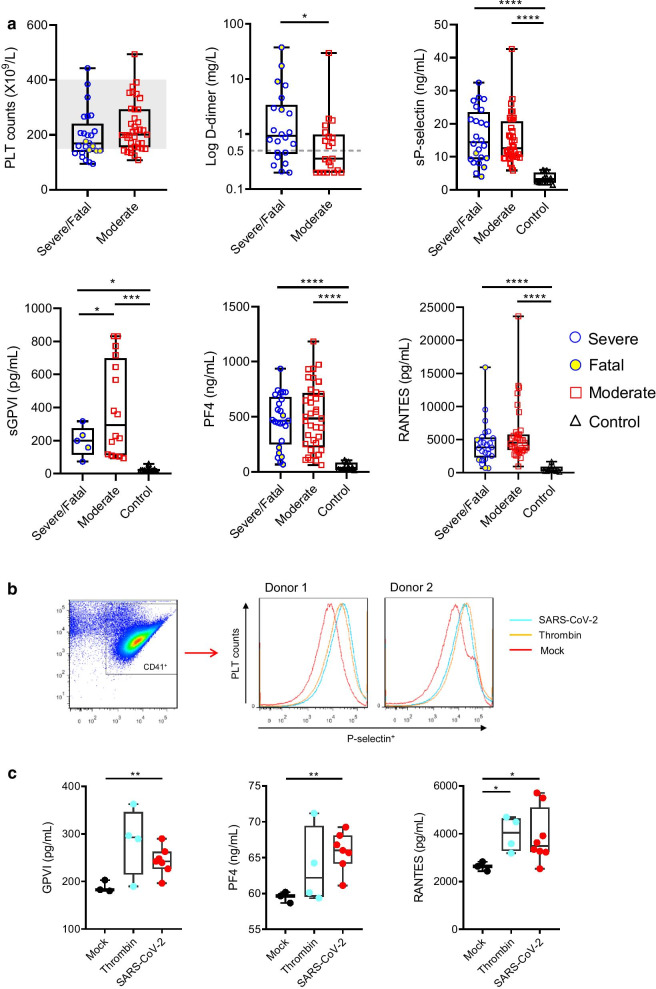
Increased platelet activation in COVID-19 patients and that stimulated by SARS-CoV-2. **a** Platelet counts and D-dimer levels of patients with severe/fatal and moderate COVID-19 are shown as the medians and interquartile ranges. The normal range of platelet count (150–400 × 10^9^/L) is shaded, and the upper limit value of D-dimer (0.5 mg/L) is indicated with dotted line. Soluble P-selectin levels (sP-selectin), soluble GPVI levels (sGPVI), PF4, and RANTES in plasma of patients with severe/fatal or moderate COVID-19, and healthy controls were measured through ELISA. **b** Platelet activation was investigated using platelets from healthy donors incubated with SARS-CoV-2, thrombin, or virus culture medium (Mock) for 3 h at 37 °C. P selectin surface translocation was measured using flow cytometry and results using platelets from two healthy donors are shown. **c** Levels of GPVI, PF4, and RANTES in the incubation supernatants were determined through ELISA. *, P < 0.05; **, P < 0.01; ***, P < 0.001; ****, P < 0.0001

Associated with coagulative disorders, COVID-19 patients have increased platelet activation and aggregation, and platelet-monocyte aggregation [1–3], which highlights the critical role of platelets in SARS-CoV-2 infection and immunopathology [4]. Consistent with previous reports [1–3], our retrospective survey of plasma samples from a cohort of 62 cases (severe or fatal and moderate COVID-19 patients, Additional file 1: Table S1) showed that COVID-19 was associated with mild thrombocytopenia (platelet count < 150 × 10^9^/L) and increased thrombosis (elevated D-dimer levels), and patients had increased platelet activation (elevated sP-selectin and sGPVI levels) and cytokine (PF4 and RANTES) release upon platelet activation (Fig. 1a). Direct interaction of SARS-CoV-2 with human platelets was suggested based on increased P-selectin translocation on platelet surface (Fig. 1b), and elevated levels of GPVI, PF4, and RANTES in platelet culture supernatants (Fig. 1c). However, the characteristics and mechanisms of the direct interaction between SARS-CoV-2 and platelets are not well elucidated, and the role of platelet receptors in the interaction remains to be clarified [4, 5].

**Fig. 2:**
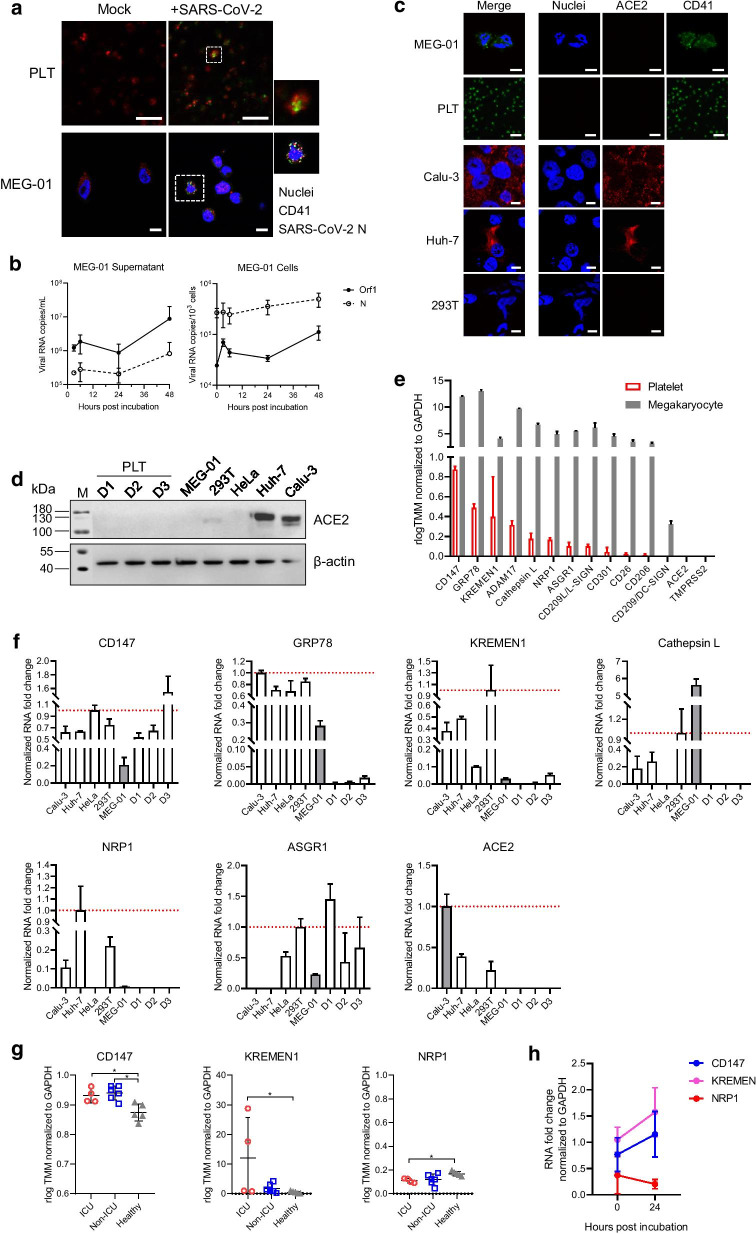
Characterization of SARS-CoV-2 interaction with human platelets and megakaryocytes. **a** IFA assays suggesting SARS-CoV-2 infection in platelets and megakaryocytes. Platelets from healthy donors and the megakaryocyte cell line MEG-01 were incubated with SARS-CoV-2 (1 MOI per test). SARS-CoV-2 N expression in platelets and MEG-01 cells were immunostained at 3 h p.i. and 24 h p.i., respectively. **b** Quantitative analysis of SARS-CoV-2 RNA copies in culture supernatants and in MEG-01 cells. **c** Immunofluorescence assay of ACE2 expression in MEG-01, platelets (PLT), Calu-3, Huh7, and 293 T cell lines. Bars, 10 μm. **d** Western blot analyses of ACE2 expression in cell lines including MEG-01, 293 T, HeLa, Huh7, and Calu-3, and platelets (PLT) from three healthy donors (D1, D2, and D3). Expression of β-actin in cells were blotted as inner control. **e** RNA transcripts of 14 receptors in human platelets and megakaryocytes were evaluated using bioinformatic methods using the RNA-seq data obtained from previous studies. **f** qRT-PCR detection of CD147, GRP78, KREMEN1, Cathepsin L, NRP1, ASGR1, and ACE2 in cell lines including Calu-3, Huh7, HeLa, 293 T, and MEG-01, and platelets from three healthy donors (D1, D2, and D3). The transcription levels were normalized to those of GAPDH in each of respective cell line or platelet samples and compared to MEG-01 or Calu-3 (shaded bars), as described in Additional file 1: Methods. **g** Comparison of CD147, KREMEN1, and NRP1 RNA levels in ICU and non-ICU COVID-19 patients with those in healthy persons using the RNA-seq data obtained from previous studies. **h** qRT-PCR detection of CD147, KREMEN1, and NRP1 transcription in MEG-01 cells after SARS-CoV-2 incubation

SARS-CoV-2 infection in human platelets and its progenitor megakaryocyte cell line MEG-01 in vitro was subsequently characterized. SARS-CoV-2 N expression was observed in some platelets and MEG-01 cells (Fig. 2a). SARS-CoV-2 RNA was detected in both culture supernatant and MEG-01 cells after SARS-CoV-2 incubation and could be maintained with a slight increase until 48 h p.i. (Fig. 2b). This suggests that SARS-CoV-2 may infect and replicate in megakaryocytes despite insufficient efficiency. However, we failed to observe any viral particles in MEG-01 cells through electron microscopy, probably because of using insufficient dose of viruses (1 MOI) for incubation, or limited infection in MEG-01 cells, as indicated by IFA images. SARS-CoV-2 RNA copies were lower in platelets (10–10^2^ copies/10^3^ cells) and culture supernatant (10^3^–10^4^ copies/mL), which diminished after 12 h (data not shown). Therefore, we speculate that platelets may not support SARS-CoV-2 replication. This echoes recent studies which have shown that SARS-CoV-2 entry in platelets may not be common in COVID-19 patients: SARS-CoV-2 RNA was detected in platelets from a few severe (2/25, 8% [2]; 2/11, 18.2% [6]) and non-severe (9/38, 23.7% [6]) patients and was not detected in platelets from patients (0/24 [7]).

The evidence of direct interaction between SARS-CoV-2 and platelets or megakaryocytes raised the concern whether ACE2 plays a role in the process. The IFA and western blot assays showed a lack of ACE2 expression in both human platelets and megakaryocytes (Fig. 2c, d). The RNA abundance of 14 receptors or co-factors including ACE2 in human platelets and megakaryocytes was subsequently inspected based on RNA-seq data reported in previous studies [2, 8] (Additional file 1: Table S2 and S3). As summarized in Fig. 2e, the abundance order in platelets was: CD147 > GRP78 > KREMEN1 > ADAM17 > cathepsin L > NRP1 > ASGR1 > CD209L/L-SIGN > CD301 > CD26 > CD206, but CD209/DC-SIGN, ACE2, and TMPRSS2 were not identified. Human megakaryocytes had similar receptor profiles, coupled with the detection of CD209/DC-SIGN. We also verified receptor abundance in MEG-01 and human platelets using qRT-PCR. In MEG-01 cells, CD147, GRP78, KREMEN1, cathepsin L, NRP1, and ASGR1 were detected, while in platelets, CD147, GRP78, KREMEN1, and ASGR1 were detected. ACE2 was not detected in MEG-01 cells or platelets (Fig. 2f). These results indicate that SARS-CoV-2 may use receptors other than ACE2 to interact with platelets or megakaryocytes.

Further analysis using the RNA-seq data showed unchanged GRP78, ADAM1, cathepsin L, GRP1, and ASGR1 abundance in platelets between ICU and non-ICU COVID-19 patients and healthy persons and revealed elevated CD147 and KREMEN1 levels and reduced NRP1 levels in patients (Fig. 2g). This was also observed in MEG-01 cells with increased CD147 and KREMEN1 levels and slightly reduced NRP1 levels after SARS-CoV-2 incubation (Fig. 2h). These data suggest that SARS-CoV-2 infection may alter gene transcription in platelets and megakaryocytes, which is similar to DENV infection that markedly changes the platelet and megakaryocyte transcriptome [8].

Owing to their roles in binding to spike protein and facilitating virus entry [9–11], CD147, KREMEN1, and NRP1 triggering of SARS-CoV-2 entry in human platelets and megakaryocytes requires in-depth investigation. Moreover, based on the original functions of CD147 in signaling pathways via cell–cell interactions [9] and of NRP1 in cardiovascular, neuronal, and immune systems [10], SARS-CoV-2 interaction with platelets is suspected to regulate platelet-mediated immune response [12] and promote coagulation dysfunction in COVID-19 [10].

## Supplementary Information

**Additional file 1.** Detailed materials and methods, and supplementary tables and figure.

## Data Availability

All data generated or analyzed during this study are included in this published article (and its supplementary information files).

## Abbreviations

COVID-19: Coronavirus disease 2019
SARS-CoV-2: Severe acute respiratory syndrome coronavirus
SARS-CoV-2 N: SARS-CoV-2 nucleoprotein
ACE2: Angiotensin-converting enzyme 2
sP-selectin: Soluble P-selectin
sGPVI: Soluble glycoprotein VI
PF4: Platelet factor 4
RANTES: C-C motif chemokine ligand 5, CXCL5
CD147: *Basigin*
GRP78: Glucose regulating protein 78
KREMEN1: Kringle containing transmembrane protein 1
ADAM17: A disintegrin and metalloproteinase 17
NRP1: Neuroplin-1
ASGR1: Asialoglycoprotein receptor 1
CD209L/L-SIGN: C-type lectin domain family 4, member M, CLEC4M
CD301: C-type lectin domain containing 10A, CLEC10A
CD26: Dipeptidyl peptidase 4, DPP4
CD206: Macrophage mannose receptor, MMR
CD209/DC-SIGN: Dendritic cell (DC)-specific intracellular adhesion molecule 3 (ICAM-3)-grabbing non-integrin
TMPRSS2: Transmembrane serine protease 2
DENV: Dengue virus
IFAs: Immunofluorescence assays
qRT-PCR: Quantitative reverse transcription-polymerase chain reaction
MOI: Multiplicity of infection
h p.i.: Hours post-inoculation
