# Supplementary material for: SARS-CoV-2 interacts with platelets and megakaryocytes via ACE2-independent mechanism
Source: J Hematol Oncol. 2021 Apr 29;14:72. doi: 10.1186/s13045-021-01082-6 (PMC8082485; doi:10.1186/s13045-021-01082-6)
Supplement: Supplementary file 1 — Additional file 1. Detailed materials and methods, and supplementary tables and figure. [file 13045_2021_1082_MOESM1_ESM.docx]

**Supplemental material**

**Materials and Methods**

*Plasma collection from COVID-19 patients and healthy donors*

COVID-19 patients (N=62) hospitalized in the Union Hospital of Huazhong University of Science and Technology (Wuhan, China) in January and February 2020 were recruited for this study. SARS-CoV-2 infection was confirmed using reverse transcription polymerase chain reaction according to the guidelines for diagnosis and treatment of COVID-19 issued by the National Health Commission of the People’s Republic of China (NHC, PRC). Disease severity was classified as mild, moderate, or severe, according to the definitions specified in the Guidance of Diagnosis and Treatment for COVID-19 issued by the National Health Commission of China. Thirty-seven of the 62 COVID-19 patients had moderate disease presenting with common symptoms including respiratory infection and typical abnormalities including viral pneumonia based on tomography. Moreover, 25 patients, including five fatal cases, had severe disease with respiratory failure and required mechanical ventilation and additionally met at least one of the following conditions: respiratory rate (RR) ≥30/min, oxygen saturation ≤93% at resting state, or PaO_2_/FiO_2_ ratio ≤300 mmHg (Supplementary Table S1). Plasma samples were obtained by collecting blood from COVID-19 patients or healthy donors in vacuum tubes containing ethylene diamine tetraacetic acid and the samples were centrifuged at 1500×*g* for 20 min. All samples were stored at −80 °C until further examination.

*Platelet isolation from healthy donors*

Platelets from healthy donors were isolated from venous blood collected in vacuum tubes containing acid-citrate-dextrose and were purified with slight modifications as previously described [1]. Briefly, blood was centrifuged at 200×*g* for 20 min. Seventy-five percent of the top platelet-rich plasma layer was removed and centrifuged at 200×*g* in the presence of 100 nM prostaglandin E1 (PGE1; Cayman Chemical, Ann Arbor, MI, USA, catalog # 13010) for another 20 min. Pellets were resuspended in PIPES buffer (145 mM NaCl, 4 mM KCl, 0.5 mM Na_2_HPO_4_, 1 mM MgCl_2_·6H_2_O, 5 mM PIPES, 5.5 mM glucose, pH 7.0) containing 100 nM PGE1, and centrifuged at 800×*g* for 5 min. Platelets were resuspended in Medium 199 (M199, Sigma, Saint Louis, MO, USA; catalog # M4530), and their purity (>98% CD41^+^) was confirmed via flow cytometry.

*Cells and virus*

Human lung epithelial cells (Calu-3; ATCC: HTB-55) and human cervix adenocarcinoma (HeLa; ATCC: CCL-2) were maintained in Eagle's minimum essential medium (EMEM; NovaStar, Wuhan, China) containing 10% fetal bovine serum (FBS; Gibco, Grand Island, NY, USA). Human hepatocarcinoma cells (Huh7; NVRC: IVCAS 9.005) and kidney epithelial cells (293T; ATCC: CRL-11268) were maintained in Dulbecco’s modified Eagle’s medium (DMEM; NovaStar, Wuhan, China) supplemented with 10% FBS. Human megakaryoblasts (MEG-01; ATCC: CRL-2021) were maintained in RPMI-1640 medium (Gibco) containing 10% FBS. SARS-CoV-2 (strain BetaCoV/Wuhan/WIV04/2019) was preserved in the National Virus Resource Center (NVRC: IVCAS 6.7512).

*Detection of platelet activation using ELISA*

Levels of soluble P-selectin (sP-selectin) and soluble glycoprotein VI (sGPVI) secreted into the plasma of COVID-19 patients and culture supernatants of platelets after SARS-CoV-2 incubation were quantified using commercially available ELISA kits (Cusabio, Wuhan, China, catalog #s CSB-E04708h and CSB-E10117h). Platelet factor 4 (PF4/CXCL4) and chemokine ligand 5 (RANTES/CCL5) were quantified using commercially available ELISA kits (R&D Systems, Minneapolis, MN, USA, catalog #s DPF40 and DRN00B).

*Platelet and megakaryocyte stimulation in vitro by SARS-CoV-2*

Purified platelets from healthy donors or the megakaryocyte cell line MEG-01 were incubated with SARS-CoV-2 (1 MOI per test), thrombin (0.5 U/mL; Cayman; catalog # 13188), or culture medium from mock-infected Vero E6 cells (ATCC; CRL-1586) for 1 h at 37°C. The supernatants were removed and replaced with fresh M199 or RPMI-1640 medium supplemented with 2% FBS after two washes with the respective culture medium. Levels of GPVI, PF4, and RANTES in supernatants at 3 h p.i. were quantified through ELISA using the commercial kits mentioned above, and P-selectin surface translocation was examined via flow cytometry.

Platelets (1×10^6^ per well) and MEG-01 cells (2×10^5^ per well) were incubated with SARS-CoV-2 (1 MOI per test) at 37°C for 1 h. The supernatants were removed, washed twice with medium for two times, and maintained in the corresponding culture medium at 37°C. Subsequently, the supernatants and MEG-01 cells or platelets were harvested at the indicated time points after inoculation with SARS-CoV-2. Cells were used to detect the viral antigen and ACE2 expression through immunofluorescence assays (IFAs) and to determine the number of SARS-CoV-2 RNA copies or receptor gene transcripts through qRT-PCR. Total RNA was purified from cell debris-clarified supernatants and was used to detect SARS-CoV-2 RNA through qRT-PCR.

*Flow cytometry analysis*

Platelets were incubated with FITC-conjugated anti-CD41 (Abcam, Cambridge, UK; catalog # ab21851) and PE-conjugated anti-CD62 (BD Bioscience, San Jose, US; catalog # 550561) according to the manufacturer’s instructions. Platelets were distinguished by specific binding of anti-CD41, and platelet surface P-selectin was quantified using a FACSCalibur flow cytometer (BD Biosciences, Franklin Lakes, NJ, USA).

*Immunofluorescence assay and western blot analysis*

To verify ACE2 expression, cells and platelets were fixed with 4% paraformaldehyde, permeabilized with 0.2% Triton X-100, and blocked with 5% BSA in PBS. The fixed cells were incubated with an ACE2 rabbit monoclonal antibody (Beyotime, AF2335, Shanghai, China) at 4 °C overnight. After three washes, the cells were incubated with goat anti-rabbit IgG H&L (Alexa Fluor® 555; Abcam, ab150078, USA).

To visualize SARS-CoV-2 interaction with megakaryocytes and platelets, the fixed cells and platelets were penetrated and blocked as mentioned above, and were incubated with a rabbit anti-CD41 antibody (Abcam, ab63983, USA) and mouse anti-SARS-CoV-2 N monoclonal antibody (SR24, kindly provided by Prof. Wuxiang Guan at Wuhan Institute of Virology, Chinese Academy of Science) at 4°C overnight. Then, the cells and platelets were incubated with a goat anti-mouse IgG H&L (Alexa Fluor® 488) (Abcam, ab150113, USA) and goat anti-rabbit IgG H&L (Alexa Fluor® 555) (1:5000 dilution) (Abcam, ab150078, USA) on the second day. Cell nuclei were stained with DAPI (Beyotime) according to the manufacturer’s instructions. Images were captured using a laser-scanning confocal microscope (60X, Nikon A1 MP STORM, Japan).

ACE2 expression in different cell lines and platelets was analyzed through western blotting using ACE2 rabbit monoclonal antibody and anti-ACTB mouse monoclonal antibody (Sangon Biotech, Shanghai, China) as the primary antibodies and HRP-conjugated Affinipure goat anti-rabbit IgG (H+L) (Proteintech Group, Wuhan, China) and HRP-conjugated goat anti-mouse IgG (H+L) (Proteintech Group) as the secondary antibodies.

*Quantitative detection of SARS-CoV-2 RNA and viral receptor RNA transcripts*

Total RNA was extracted from the supernatants (500 μL) and cells or platelets using QIXzol reagent (NovaStar, NZK-R15008, Wuhan, China). SARS-CoV-2 RNA copies were determined via qRT-PCR using the commercial One Step PrimeScript RT-PCR Kit (TaKaRa, RR064A, Japan) with primers and probes targeting Orf1 and N, respectively (Supplementary Table S4).

Viral receptor gene transcription in different cell lines and platelets was verified through qRT-PCR. Total RNA was purified fromCalu-3, HeLa, Huh-7, HEK293T, and MEG-01 cells and platelets from three healthy donors, and treated with Turbo^TM^ DNase (Invitrogen, AM2238, USA) to eliminate residual DNA. DNA elimination was confirmed through PCR performed using the glyceraldehyde-3-phosphate dehydrogenase (GAPDH) primers (Supplementary Table S4) and the commercial 2 × Phanta Master Mix (Vazyme, Nanjing, China) (Supplementary Figure S1). Receptor gene transcripts were detected via qRT-PCR using DNA-free RNA from cells as templates and respective primers, as listed in Table S1. Receptor abundance was quantified using the 2 ΔΔCT method by normalizing it to abundance of GAPDH and then was compared to the abundance of in MEG-01, which was set as control A for CD147, GRP78, KREMEN1, Cathepsin L, NRP1, and ASGR1. The abundance of ACE2 was normalized and compared to that of Calu-3. To better present the abundance variations of receptors between cell lines and platelets, the cell lines Calu-3, Huh-7, HeLa, and 293T, which had the highest abundance value of one receptor, were set as control B (abundance = 1), and the identical receptor of the other cell lines and platelets were normalized to the value of control B to present the RNA fold change.

*Bioinformatics analysis of RNA transcripts of SARS-CoV-2 receptors in human platelets and megakaryocytes*

Raw RNA-seq data of human megakaryocytes (PRJNA517714) and platelets from COVID-19 patients and healthy donors (PRJNA634489) were downloaded from the NCBI SRA database according to previous studies [2, 3]. After quality control using FastQC, Hisat2 and htseq-count were used to calculate the number of reads for the indicated receptor genes, as previously described [4, 5]. The standardized gene expression level was calculated using DESeq2 and presented as the rlogTMM value as previously described [6], and then normalized to the level of the housekeeping gene GAPDH.

*Receptors or co-factors for SARS-CoV-2 entry*

Fourteen receptors or co-factors related to SARS-CoV-2 entry and infection were analyzed based on literature reports, as listed below:

**ACE2:** ACE2 is the conventional cell receptor through which SARS-CoV-2 infects and enters cells [7].

**CD147:** CD147 was shown to be a novel receptor mediating SARS-CoV-2 entering host cells by endocytosis [8], and anti-CD147 invention and drugs could be an option for COVID-19 therapy [9, 10].

**GRP78:** Molecular docking and structural bioinformatics analysis suggested that SARS-CoV-2 S protein can interact with GRP78 on the surface of human respiratory cells [11].

**ASGR1 and KREMEN1:** The ectodomains of ASGR1 and KREMEN1 are sufficient to enable SARS-CoV-2 entry, and together with ACE2, ASGR1, and KREMEN1 correlate with SARS-CoV-2 multi-organ tropism, further indicating that ASGR1 and KREMEN1 are alternative entry receptors [12].

**TMPRSS2 and ADAM17:** TMPRSS2 and ADAM17 that participate in ACE2 ectodomain cleavage [13] are suggested to facilitate the uptake of SARS-CoV-2 virions into host cells [14]. TMPRSS2 can also prime SARS-CoV-2 S protein activation and affect virus spreading after virus entry is initiated by binding to ACE2 [15].

**Cathepsin L:** Cathepsin L is involved in processing SARS-CoV-2 S protein in a pH-dependent manner that mediates virus entry, and was found to be upregulated in the lung tissue of COVID-19 patients [16, 17].

**NRP-1:** NRP-1 binds to the C-end rule motif on SARS-CoV-2 S1 generated by the cleavage of the S protein to facilitate SARS-CoV-2 cell entry and infection [18, 19].

**CD209/DC-SIGN and CD209L/L-SIGN:** CD209/DC-SIGN and CD209L/L-SIGN were identified as entry receptors that mediate SARS-CoV-2 infection in endothelial cells, monocytes, and T-lymphocytes in both ACE2-dependent and -independent manners [20].

**CD206 and CD301**: CD206 and CD301 are mannose receptors highly expressed in monocytes, dendritic cells, and macrophages, and exhibit strong binding to SARS-CoV-2 S protein as well as the S1 domain in a glycan-dependent manner [21].

**CD26**: CD26 is a potential SARS-Co-2 receptor binding to the S1 domain, as proposed by *in silico* experiments [22, 23], and is suggested as a potential therapeutic target because of its inflammatory properties [24].

*Statistical analysis*

The results of continuous variables are expressed as medians with interquartile ranges. The statistical significance of differences between two groups was determined using Student’s t-test. Statistical analyses were performed using GraphPad Prism version 7 (GraphPad Software, San Diego, CA, USA). Statistical significance was set at *P*<0.05.

**References**

1. Koupenova M, Corkrey HA, Vitseva O, Manni G, Pang CJ, Clancy L, et al. The role of platelets in mediating a response to human influenza infection. Nat Commun.2019;10(1):1780.

2. Manne BK, Denorme F, Middleton EA, Portier I, Rowley JW, Stubben CJ, et al. Platelet gene expression and function in patients with COVID-19. Blood. 2020;136:1317–29.

3. Campbell RA, Schwertz H, Hottz ED, Rowley JW, Manne BK, Washington AV, et al. Human megakaryocytes possess intrinsic antiviral immunity through regulated induction of IFITM3. Blood. 2019;133(19):2013–26.

4. Anders S, Pyl PT, Huber W. HT. HTSeq--a Python framework to work with high-throughput sequencing data. Bioinformatics. 2015;31(2):166–9.

5. Kim D, Langmead B, Salzberg SL. HISAT: a fast spliced aligner with low memory requirements. Nat Methods. 2015;12(4):357–60.

6. Love MI, Huber W, Anders S. Moderated estimation of fold change and dispersion for RNA-seq data with DESeq2. Genome Biol.2014;15(12):550.

7. Zhou P, Yang XL, Wang XG, Hu B, Zhang L, Zhang W, et al. A pneumonia outbreak associated with a new coronavirus of probable bat origin. Nature. 2020;579(7798):270–3.

8. Wang K, Chen W, Zhang Z, Deng Y, Lian JQ, Du P, et al. CD147-spike protein is a novel route for SARS-CoV-2 infection to host cells. Signal Transduct Target Ther. 2020;5(1):283.

9. Radzikowska U, Ding M, Tan G, Zhakparov D, Peng Y, Wawrzyniak P, et al. Distribution of ACE2, CD147, CD26, and other SARS-CoV-2 associated molecules in tissues and immune cells in health and in asthma, COPD, obesity, hypertension, and COVID-19 risk factors. Allergy. 2020;75(11):2829–45.

10. Pourani MR, Abdollahimajd F. CD147 as a novel receptor in the pathogenesis of SARS-CoV-2: is there any correlation with the risk of COVID-19 in dermatological diseases? Dermatol Ther.2020;33(6):e14443.

11. Ibrahim IM, Abdelmalek DH, Elshahat ME, Elfiky AA. COVID-19 spike-host cell receptor GRP78 binding site prediction. J Infect.2020;80(5):554–62

12. Gu Y, Cao J, Zhang X, Gao H, Wang Y, Wang J, et al. Interaction network of SARS-CoV-2 with host receptome through spike protein; bioRxiv-Microbiol; 2020. doi: [10.1101/2020.09.09.287508](https://doi.org/10.1101/2020.09.09.287508)

13. Heurich A, Hofmann-Winkler H, Gierer S, Liepold T, Jahn O, Pöhlmann S. TMPRSS2 and ADAM17 cleave ACE2 differentially and only proteolysis by TMPRSS2 augments entry driven by the severe acute respiratory syndrome coronavirus spike protein. J Virol.2014;88(2):1293–307.

14. Aguiar JA, Tremblay BJ, Mansfield MJ, Woody O, Lobb B, Banerjee A, et al. Gene expression and *in situ* protein profiling of candidate SARS-CoV-2 receptors in human airway epithelial cells and lung tissue. Eur Respir J.2020;56(3):2001123

15. Hoffmann M, Kleine-Weber H, Schroeder S, Krüger N, Herrler T, Erichsen S, et al.SARS-CoV-2 cell entry depends on ACE2 and TMPRSS2 and is blocked by a clinically proven protease inhibitor. Cell. 2020;181(2):271–280.e8.

16. Gomes CP, Fernandes DE, Casimiro F, da Mata GF, Passos MT, Varela P, et al. Cathepsin L in COVID-19: from pharmacological evidences to genetics. Front Cell Infect Microbiol.2020;10:589505.

17. Padmanabhan P, Desikan R, Dixit NM. Targeting TMPRSS2 and cathepsin B/L together may be synergistic against SARS-CoV-2 infection. PLOS Comput Biol.2020;16(12):e1008461.

18. Cantuti-Castelvetri L, Ojha R, Pedro LD, Djannatian M, Franz J, Kuivanen S, et al. Neuropilin-1 facilitates SARS-CoV-2 cell entry and infectivity. Science. 2020;370(6518):856–60.

19. Daly JL, Simonetti B, Klein K, Chen KE, Williamson MK, Antón-Plágaro C et al. Neuropilin-1 is a host factor for SARS-CoV-2 infection. Science. 2020;370(6518):861–5.

20. Amraie R, Napoleon MA, Yin W, Berrigan J, Suder E, Zhao G, et al. CD209L/L-SIGN and CD209/DC-SIGN act as receptors for SARS-CoV-2 and are differentially expressed in lung and kidney epithelial and endothelial cells. bioRxiv. 2020. doi: [10.1101/2020.06.22.165803](https://doi.org/10.1101/2020.06.22.165803)

21. Gao C, Zeng J, Jia N, Stavenhagen K, Matsumoto Y, Zhang H, et al.SARS-CoV-2 spike protein interacts with multiple innate immune receptors. bioRxiv. 2020. doi: [10.1101/2020.07.29.227462](https://doi.org/10.1101/2020.07.29.227462)

22. Vankadari N, Wilce JA. Emerging WuHan (COVID-19) coronavirus: glycan shield and structure prediction of spike glycoprotein and its interaction with human CD26. Emerg Microbes Infect.2020;9(1):601–4.

23. Raha AA, Chakraborty S, Henderson J, Mukaetova-Ladinska E, Zaman S, Trowsdale J, et al. Investigation of CD26, a potential SARS-CoV-2 receptor, as a biomarker of age and pathology. Biosci Rep.2020;40(12):BSR20203092.

24. Valencia I, Peiró C, Lorenzo Ó, Sánchez-Ferrer CF, Eckel J, Romacho T. DPP4 and ACE2 in Diabetes and COVID-19: Therapeutic Targets for Cardiovascular Complications? Front Pharmacol.2020;11:1161.

**Supplementary tables**

**Supplementary Table S1** Personal information, comorbidities, and laboratory findings of COVID-19 patients and healthy donors recruited in this study

| Characteristics | Control (n=14) | COVID-19 Patients (n=62) | Severe/Fatal (n=25) | Moderate  (n=37) |
| --- | --- | --- | --- | --- |
| Age, median year (IQR) | 32.5 (27-37.5) | 66 (60-71) | 68 (62-75) | 66 (56-71) |
| Sex, n female/male (%) | 8/6 (57.1/42.9) | 30/32  (48.4/51.6) | 15/10 (60.0/40.0) | 17/20 (45.9/54.1) |
| Survival, n (%) | N/A | 57 (91.9) | 20 (80.0) | 37 (100.0) |
| Comorbidities ^a^ |  |  |  |  |
| Hypertension, n/records (%) | 1 (12.5) | 20/55 (40.0) | 11/21 (52.4) | 9/34 (26.5) |
| Diabetes, n/records (%) | 0 | 7/55 (12.7) | 5/21 (23.8) | 2/34 (5.9) |
| Laboratory findings | | | | |
| PLT count (×10^9^/L) ^b^, median (IQR) | N/A | 198 (149-264) | 169 (142-213) | 201 (156-293) |
| <150, n (%) | N/A | 16 (25.8) | 9 (36.0) | 7 (18.9) |
| D-dimer (mg/L) ^c^, median (IQR) | N/A | 0.74 (0.28-1.605) | 0.925 (0.46-2.9125) | 0.36 (0.2-0.89) |
| >0.5, n (%) | N/A | 25 (58.1) | 15 (68.2) | 10 (47.6) |

^a^, Comorbidities were summarized based on 55 of the 62 patients with comorbidity records (21 severe/fatal and 34 moderate).

^b^, The normal range of platelet count refers to 150-400 ×10^9^/L.

^c^, D-dimer levels in plasma were not tested for each of the 62 patients. The data were analyzed based on 43 of the 62 patients who had D-dimer level tests (22 severe/fatal and 21 moderate).

IQR, interquartile range; PLT, platelet; N/A, not applicable.

**Supplementary Table S2** The number of reads of the receptor genes in platelets from healthy donors and COVID-19 patients obtained from the transcriptome data of a previous study (ref 2)

| Receptor | Reads (mean ± SD) in platelets | | |
| --- | --- | --- | --- |
|  | ICU (n=4) | Non-ICU (n=6) | Healthy (n=5) |
| ACE2 | 0 | 0 | 0 |
| TMPRSS2 | 0 | 0.17±0.41 | 0 |
| Cathepsin L | 26.50±39.57 | 15.50±16.74 | 3.40±5.64 |
| CD209L/L-SIGN | 0.25±0.50 | 2.17±4.36 | 0.6±1.34 |
| CD209/DC-SIGN | 0 | 0.17±0.41 | 0.2±0.45 |
| ADAM17 | 43.25±15.19 | 49±37.78 | 11.80±11.32 |
| CD147 | 19562.00±5559.52 | 13931.67±2567.22 | 1965.80±380.11 |
| GRP78 | 450.00±357.01 | 483.83±404.29 | 52.40±40.50 |
| CD26 | 1.25±1.89 | 1.67±2.73 | 1.00±0.71 |
| MR/CD206 | 2±3.67 | 1±0.89 | 0.8±0.84 |
| MGL/CLEC10A/CD301 | 0.25±0.5 | 0.33±0.52 | 2.8±3.36 |
| ASGR1 | 5.25±5.12 | 4.5±2.59 | 2±3.46 |
| KREMEN1 | 223.75±231.27 | 47.5±53.48 | 2.4±2.88 |
| NRP1 | 2.5±2.38 | 9.17±8.57 | 3.6±2.07 |
| GAPDH | 36945.5±4375.10 | 23636.33±7060.92 | 8161.4±3227.19 |

**Supplementary Table S3** The number of reads of the receptor genes in megakaryocytes from healthy donors obtained from the transcriptome data of a previous study (ref 3)

| Gene | Reads in megakaryocytes |
| --- | --- |
| ACE2 | 0 |
| TMPRSS2 | 0 |
| Cathepsin L | 77.7±8.3 |
| CD209L/L-SIGN | 9.3±6.4 |
| CD209/DC-SIGN | 1±0 |
| ADAM17 | 859±319 |
| CD147 | 4123.7±1208.9 |
| GRP78 | 8935.7±3094.4 |
| CD26 | 12.3±9.7 |
| MR/CD206 | 8.3±3.1 |
| MGL/CLEC10A/CD301 | 20.7±16 |
| ASGR1 | 46.3±11.0 |
| Kremen1 | 15±3 |
| NRP1 | 29.3±28.4 |
| GAPDH | 21322±8146.9 |

**Supplementary Table S4** Primers used for qRT-PCR detection of receptors, co-factors, and viral RNA

| Gene | Sequences (5′-3′) | | Gene accession No. | Product (bp) |
| --- | --- | --- | --- | --- |
| CD147 | For: | CTCCGACCAGGCCATCATCAC | NM_198590.3 | 129 |
|  | Rev: | GGCTTCCGGCGCTTCTCGTA |  |  |
| NPC1 | For: | AAGTGACAAAGGAACGGCTTGG | XM_005258277.1 | 106 |
|  | Rev: | GCCCTCAAATGCTGCGCTGA |  |  |
| GRP78 | For: | GCCCAACGCCAAGCAACCAA | NM_005347.5 | 91 |
|  | Rev: | AAGCAATAGCAGCTGCCGTAG |  |  |
| KREMEN1 | For: | CCCGAGTGTTTCACAGCCAAT | NM_001039570.3 | 107 |
|  | Rev: | GGATGCTGGAAAGTCTCGTTC |  |  |
| NRP1 | For: | CCTCACATTGGGCGTTACTGTGGA | NM_003873.7 | 189 |
|  | Rev: | TCCTGATTCCATGCCCAGAGCTTC |  |  |
| Cathepsin L | For: | TGGTAGACTGCTCTGGGCCTC | NM_001382757.1 | 97 |
|  | Rev: | GAGTCCAGGCCTCCATTATCCTG |  |  |
| ACE2 | For: | TTATTGGAGAGGAGACTA | NM_001371415.1 | 168 |
|  | Rev: | CTGATATAGGAAGGATAGG |  |  |
| ASGR1 | For: | AGCGCAGCTGCTACTGGTTC | NM_001671.5 | 112 |
|  | Rev: | TTCTGCTCCTCCCAGGACGT |  |  |
| GAPDH | For: | CAAGGGCATCCTGGGCTACACT | NM_002046.7 | 85 |
|  | Rev: | CCCAGCGTCAAAGGTGGAGGA |  |  |
| SARS-CoV-2 N | For: | TAACCAGAATGGAGAACGCAGTG | NC_045512.2 | 96 |
|  | Rev: | TGAGTGAGAGCGGTGAACCAAGAC |  |  |
|  | Probe: | HEX-ATCAAAACAACGTCGGCCCCAAGGT-BHQ2 |  |  |
| SARS-CoV-2 Orf1 | For: | CAAAATGYTGGACTGAGACTGACC | NC_045512.2 | 154 |
|  | Rev: | ACGATATCATCDACAAAACAGCCG |  |  |
|  | Probe: | FAM-ATCTGGGTAAGGMAGGTACACRTAATCATCAC-BHQ1 |  |  |

**Supplementary Figure**

**Supplementary Figure S1.** PCR detection of total RNA from cell lines and human platelets. Total RNA purified from human cell lines and platelets from healthy donors (D1, D2, and D3) was treated with or without DNase to remove residual DNA. PCR was performed using GAPDH primers to confirm DNA elimination in each sample. M, DNA marker; NC, negative control.
